# The PCK2‐Traf6‐Tollip Axis Restricts PEDV Replication by Orchestrating Selective Autophagic Degradation of the Viral Nucleocapsid Protein

**DOI:** 10.1155/tbed/8776901

**Published:** 2026-06-29

**Authors:** Ao Gao, Xinyu Yang, Wenzhen Qin, Dongfang Zheng, Yuchang Liu, He Sun, Zongyan Chen, Guangzhi Tong, Shuang Xue, Ning Kong, Lanlan Zheng

**Affiliations:** ^1^ College of Veterinary Medicine, Henan Agricultural University, Zhengzhou, 450046, China, henau.edu.cn; ^2^ Shanghai Veterinary Research Institute, Chinese Academy of Agricultural Sciences, Shanghai, China, caas.cn; ^3^ Longhu Laboratory of Henan Province, Zhengzhou, 450046, China; ^4^ Zhumadian Animal Disease Control and Inspection Center, Zhumadian, Henan, China; ^5^ Jiangsu Co-innovation Center for Prevention and Control of Important Animal Infectious Diseases and Zoonoses, Yangzhou University, Yangzhou, 225009, China, yzu.edu.cn

**Keywords:** autophagy pathway, degradation, PCK2, PEDV N

## Abstract

Porcine epidemic diarrhea virus (PEDV) is a devastating enteric pathogen in neonatal piglets, causing outbreaks with high mortality. The lack of effective vaccines or treatments makes elucidating host‐pathogen interactions essential for developing control strategies. In this study, we identify phosphoenolpyruvate carboxykinase 2 (PCK2) as a host restriction factor that targets the viral nucleocapsid (N) protein for degradation, thereby suppressing PEDV replication. This PCK2‐mediated antiviral effect was reversed by autophagy inhibitors, indicating the involvement of a selective autophagic. Mechanistically, we found that PCK2, the N protein, the E3 ubiquitin ligase Traf6, and the cargo receptor Tollip form a functional complex. Depletion of either Traf6 or Tollip disrupted the autophagy pathway, restored N protein stability, and consequently rescued viral replication from PCK2 inhibition. This study unveils a novel antiviral mechanism in which PCK2 orchestrates the selective autophagic degradation of the PEDV N protein, highlighting the PCK2‐Traf6‐Tollip axis as a promising therapeutic direction.

## 1. Introduction

Porcine epidemic diarrhea virus (PEDV) is a virus belonging to the genus *Alphacoronavirus* of the family *Coronaviridae* in the order *Nidovirales*. It was first discovered in the United Kingdom [1] and Belgium [2] in 1971. This disease has been present in the pig farming industries in Europe and Asia since its discovery. It spreads rapidly and causes infectious intestinal diseases characterized by vomiting, diarrhea, and dehydration, with high lethality to newborn piglets [3, 4]. Before 2010, PEDV was endemic in China’s swine population, causing only sporadic, geographically limited incidents. A transformative shift began with an outbreak in October 2010. Originating in the south and characterized by severe acute diarrhea, the disease achieved nationwide transmission in a short period [5, 6]. The ensuing epidemic had a catastrophic impact, driving mortality in piglets to 80%–100% and inflicting profound damage on the pig production department [7, 8].

The causative agent, PEDV, is characterized by a ~28 kb positive‐sense single‐stranded RNA genome (excluding the polyA tail). The genomic architecture follows a canonical order: a 5′ cap, 5′ UTR, seven ORFs, 3′ UTR, and a 3′ polyA tail [9]. Translation of the genome yields four structural proteins: spike (S), membrane (M), envelope (E), and nucleocapsid (N) [10, 11]. The N protein, weighing ~58 kDa, constitutes the predominant structural component of the virion and is indispensable for key processes such as viral replication, transcription, and translation [12]. Previous studies have reported that the N protein can prolong the S phase of the cell cycle and induce endoplasmic reticulum stress [13]. Additionally, the N protein can interact with the nucleolar phosphoprotein nucleophosmin (NPM1), preventing its hydrolysis, thereby extending cell viability and promoting PEDV proliferation [14]. Furthermore, the PEDV N protein can inhibit the production of IFN‐ and interferon‐stimulated genes by suppressing the activity of IRF3 [15]. Two N‐terminal epitopes within the PEDV N protein contribute to cross‐reactivity between PEDV and porcine transmissible gastroenteritis virus (TGEV) [16], providing critical insights for the development of precise diagnostic tools for porcine coronaviruses [17]. Due to high levels of antibodies being produced against N protein at the early stages of PEDV infection, the N protein is often utilized as an ideal target for the early diagnosis of PEDV [18].

As a fundamental degradation pathway in eukaryotic cells, autophagy is essential for maintaining cellular physiology [19]. This process initiates with the encapsulation of cytoplasmic components by double‐membraned autophagosomes, which subsequently fuse with lysosomes for the breakdown of entrapped materials into basic molecules. Autophagy facilitates adaptation to metabolic stress, removal of harmful agents (e.g., protein aggregates, damaged organelles, and pathogens), and supports development and genomic integrity [20]. A key antiviral function of autophagy involves the selective degradation of viral proteins [21]. This is achieved when E3 ubiquitin ligases, such as MARCH8, tag viral proteins like the coronavirus N protein with ubiquitin. The ubiquitinated substrates are then identified by specific cargo receptors, including NDP52, which directs them to autophagosomes for destruction, thereby inhibiting viral replication [22, 23].

Phosphoenolpyruvate carboxykinase (PCK), an evolutionarily conserved enzyme, is a critical regulator of gluconeogenesis. It exists in two isoforms in vertebrates: cytosolic PCK1 and mitochondrial PCK 2 (PCK2). The latter, PCK2, serves as the predominant isoform in the pancreas. Here, it is essential for glucose homeostasis, primarily by promoting insulin secretion and facilitating glucose sensing in pancreatic cells [24, 25]. This function allows the pancreas to integrate neuroendocrine signals from both the brain and the gut, coordinating the secretion of insulin and other digestive enzymes [26]. PCK2 utilizes mitochondrial GTP (mtGTP) as a phosphate donor to catalyze the conversion of oxaloacetate (OAA) into phosphoenolpyruvate (PEP). It serves as a crucial mechanism for recycling mtGTP produced by the succinyl‐CoA synthetase reaction [27]. Beyond its role in metabolic synthesis, PCK2 also modulates the autophagy process. Overexpression of PCK2 has been shown to promote cellular autophagy, although its critical role in this process remains unclear [28]. Based on previous research findings, we employed laboratory proteomics approaches to screen a panel of potential host factors capable of inhibiting PEDV. Furthermore, preliminary experiments validated the inhibitory effect of PCK2 on viral replication.

This study elucidates a novel antiviral mechanism by which PCK2 suppresses PEDV replication. Specifically, PCK2 facilitates the degradation of the viral N protein through the autophagy pathway, thereby inhibiting PEDV propagation.

## 2. Material and Methods

### 2.1. Antibodies and Reagents

Key reagents and their sources are listed: anti‐Flag‐tag antibody (F1804), chloroquine phosphate (CQ; PHR1258), 3‐methyladenine (3‐MA; M9281), and MG132 (M7449) (Sigma–Aldrich); antibodies for ACTB/β‐actin (66009‐1‐lg), GST‐tag (10000‐0‐AP), HRP‐labeled antimouse (SA00001‐1), and antirabbit (SA00001‐2) IgG (Proteintech Group); bafilomycin A1 (Baf A1; 54645) and anti‐HA‐tag antibody (3724) (Cell Signaling Technology). The monoclonal anti‐PEDV N antibody was previously stocked in our laboratory [29].

### 2.2. Cells and Viruses

African green monkey kidney cells (Vero cells; ATCC CCL‐81) and human embryonic kidney cells (HEK 293T; ATCC CRL‐11268) were maintained in Dulbecco’s modified Eagle medium (DMEM; Invitrogen, 12430054) supplemented with 10% fetal bovine serum (FBS; Gibco, 10099141). Porcine kidney cells (LLC‐PK1) were kindly provided by Dr. Rui Luo (Huazhong Agricultural University, Wuhan, China) and cultured in modified Eagle medium (MEM; Invitrogen, 11095080). All cell lines were incubated at 37°C under 5% CO_2_. The variant PEDV JS‐2013 strain, isolated and stored in our laboratory [30], was propagated and titrated in Vero cells, with viral titers determined using Karber’s method [29].

### 2.3. Western Blotting

Cell lysates were prepared by RIPA lysis and extraction buffer (89901, Thermo Fisher Scientific) with a protease inhibitor (B14001, Bimake). Following treatment of cell samples, a 5× SDS loading buffer was added, and the mixture was boiled for 10 min. The proteins were then separated using SDS‐PAGE electrophoresis and transferred onto a nitrocellulose M (10600001, GE Healthcare). The M was incubated with primary and secondary antibodies. Finally, enhanced chemiluminescence detection (Share‐bio, SB‐WB012) was employed to visualize protein bands.

### 2.4. Coimmunoprecipitation Assay

Posttransfection with the specified plasmids, cells were lysed in NP40 buffer and centrifuged. The cleared supernatants were incubated with anti‐Flag antibody‐conjugated Dynabeads Protein G (Life Technologies, 10004D) for immunoprecipitation. Protein complexes were eluted with 50 mM glycine elution buffer (pH 2.8) and analyzed by western blot, respectively.

### 2.5. Quantitative Real‐Time PCR (qRT‐PCR)

According to the manufacturer’s instructions, total RNA was extracted using either the RNeasy Mini Kit (Qiagen, 74104) or the QIAamp Viral RNA Mini Kit (Qiagen, 52906). The resulting RNA was then reverse‐transcribed into cDNA with the PrimeScript RT Reagent Kit (Takara, RRO47A). Gene expression levels were quantified by qRT‐PCR on a real‐time PCR system using SYBR Premix Ex Taq (Vazyme Biotech Co., Ltd., q711‐03), normalizing to β‐actin as the reference gene. The corresponding primer sequences are listed in Table 1.

**Table 1:** Oligonucleotides used in the real‐time PCR and siRNA experiments.

Purpose	Names	Sequences (5′–3′)
Real‐time PCR	PEDV N forward	GAGGGTGTTTTCTGGGTTG
	PEDV N reverse	CGTGAAGTAGGAGGTGTGTTAG
	PCK2 forward	ATGGCCGCTATGTACCGC
	PCK2 reverse	TCACATTCTGCGCACGCG
	ACTB forward	TCCCTGGAGAAGAGCTACGA
	ACTB reverse	AGCACTGTGTTGGCGTACAG
siRNA	si‐Tollip sense	GACUCUUUCUAUCUCGAGATT
	si‐Tollip antisense	UCUCGAGAUAGAAAGAGUCTT
	si‐Traf6 sense	GCGCUGUGCAAACUAUAUATT
	si‐Traf6 antisense	UAUAUAGUUUGCACAGCGCTT
	NC sense	UUCUCCGAACGUGUCACGUTT
	NC antisense	ACGUGACACGUUCGGAGAATT

### 2.6. GST Affinity–Isolation Assay

For recombinant expression in BL21‐competent cells (Vazyme Biotech, C504‐03), the coding sequences for PCK2, Traf6, Tollip, and the PEDV N protein were cloned into pCold TF or pCold GST vectors. The expressed recombinant proteins were harvested from bacterial cultures via ultrasonic lysis. Protein–protein interactions were subsequently assessed using commercial GST Protein Interaction Pull‐Down Kits (Thermo, 21516), following the manufacturer’s protocol.

### 2.7. Confocal Immunofluorescence Assay

HeLa cells were transfected with indicated plasmids, fixed with 4% paraformaldehyde (Sigma–Aldrich, P6148), and permeabilized with 0.1% Triton X‐100 (Sigma–Aldrich, T9284). Following blocking with 5% bovine serum albumin (Cell Signaling Technology, 9998), the cells were incubated with primary antibodies. Subsequently, fluorescence‐labeled secondary antibodies were applied to the cells under light‐protected conditions. Finally, the nuclei were specifically stained with DAPI. Fluorescence images were acquired using a laser scanning confocal microscope (Carl Zeiss, Oberkochen, Germany).

### 2.8. Statistical Analysis

Statistical analyses were performed with GraphPad Prism 8.0. Quantitative data are expressed as mean ± standard deviation (SD) from three independent biological replicates. Intergroup differences were assessed by two‐tailed Student’s *t*‐test, with significance thresholds defined as:  ^∗^ *p* < 0.05,  ^∗∗^ *p* < 0.01, and  ^∗∗∗^ *p* < 0.001; ns denotes nonsignificant differences (*p* ≥ 0.05).

## 3. Results

### 3.1. Overexpression of PCK2 Inhibits PEDV Infection

**Figure 1:**
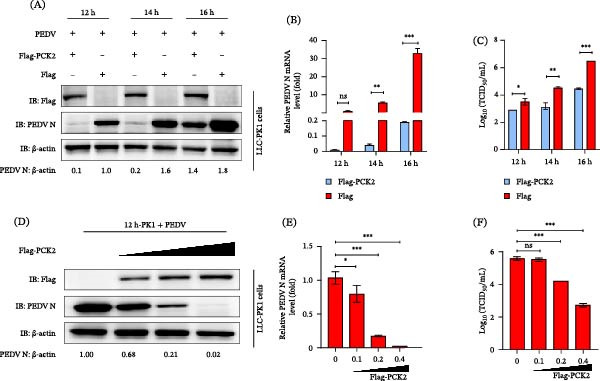
PCK2 inhibited PEDV replication. (A–C) LLC‐PK1 cells were transfected with Flag‐PCK2 plasmid and subsequently infected with PEDV (MOI = 0.01). Cell lysates and supernatants were subjected to western blotting, qRT‐PCR, and TCID_50_ assays. β‐Actin was used as a loading control. (D–F) LLC‐PK1 cells were transfected with a gradient concentration of PCK2 plasmid and infected with PEDV at an MOI of 0.01. PEDV titers were determined by western blot, qRT‐PCR, and TCID_50_ assays. Data are presented as the mean ± SD of three independent replicates.  ^∗^ *p* < 0.05,  ^∗∗^ *p* < 0.01, and  ^∗∗∗^ *p* < 0.001 (two‐tailed Student’s *t*‐test).

LLC‐PK1 cells were transfected with either a PCK2‐overexpressing plasmid or an empty vector control. At 24 h posttransfection, the cells were challenged with PEDV at an MOI of 0.01. Cell lysates and culture supernatants were harvested at 12, 14, and 16 h postinfection (hpi). Viral replication was concomitantly assessed by western blot analysis for the N protein (Figure 1A), qRT‐PCR for viral mRNA (Figure 1B), and TCID_50_ assay for infectious virus titers (Figure 1C). Results from all three methods demonstrated that PCK2 overexpression significantly suppressed the levels of PEDV N protein, viral mRNA, and infectious virus production compared to the control. Moreover, a negative correlation was observed between PCK2 expression and viral replication efficiency (Figure 1D–F). Collectively, these data indicate that PCK2 exerts an inhibitory effect on PEDV replication in LLC‐PK1 cells.

### 3.2. PCK2 Targets and Degrades PEDV N Protein Through the Autophagy Pathway

**Figure 2:**
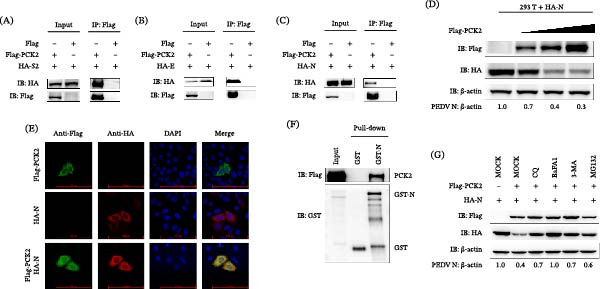
PCK2 targets and degrades the N protein via autophagy. (A–C) HEK 293T cells were cotransfected with HA‐S2, HA‐E, and HA‐N plasmids along with the Flag‐PCK2 plasmid. Co‐IP assay was performed using anti‐Flag beads, and the precipitated proteins were analyzed by western blot. (D) HEK 293T cells were cotransfected with a gradient concentration of PCK2 plasmid and a constant concentration of HA‐N plasmid. Cell lysates were analyzed by immunoblotting. (E) HeLa cells were cotransfected with HA‐N and Flag‐PCK2 plasmids and labeled with corresponding antibodies. Fluorescence signals were observed under a confocal immunofluorescence microscope (scale bar = 100 µm). (F) GST affinity purification assay was used to analyze the interaction between PCK2 and N protein. (G) HEK 293T cells were cotransfected with Flag‐PCK24 and HA‐N plasmids and treated with CQ, Baf A1, 3‐MA, and MG132. Cell lysates were analyzed by immunoblotting.

To elucidate the mechanism by which PCK2 inhibited PEDV replication, the interactions between PCK2 and PEDV structural proteins (S1, S2, E, and N) were investigated. HEK 293T cells were cotransfected with plasmids coding the PEDV structural protein and PCK2 protein, followed by Co‐IP analysis. It was observed that Flag‐PCK2 coprecipitated with PEDV S2, E, and N proteins (Figure 2A–C), with the exception of S1 (Figure S1A). Additionally, overexpression of PCK2 reduced the levels of the N protein in a dose‐dependent manner (Figure 2D), not S2 or E protein (Figure S1B, C), suggesting that PCK2 might be degrading the N protein. GST pull‐down and confocal immunofluorescence assays further confirmed the binding between PEDV N and PCK2 (Figure 2E,F). To determine whether the N protein undergoes proteasomal degradation or autophagic‐lysosomal hydrolysis, plasmids encoding PEDV N and PCK2 were cotransfected into HEK 293T cells, and the cells were treated with the proteasome inhibitor MG132 and autophagy inhibitors 3‐MA, CQ, or Baf A1. Western blot assay showed that autophagy inhibitors 3‐MA, CQ, and Baf A1 blocked PCK2‐mediated degradation of the N protein, whereas the proteasome inhibitor MG132 had no significant effect (Figure 2G). Therefore, the degradation of the N protein was likely mediated through the autophagic‐lysosomal pathway. These results suggested that PCK2 interacted with the PEDV N protein and promoted its degradation via autophagy [31].

### 3.3. Traf6 Ubiquitinates the PEDV N Protein and Promotes PCK2‐Mediated Degradation of the N Protein

**Figure 3:**
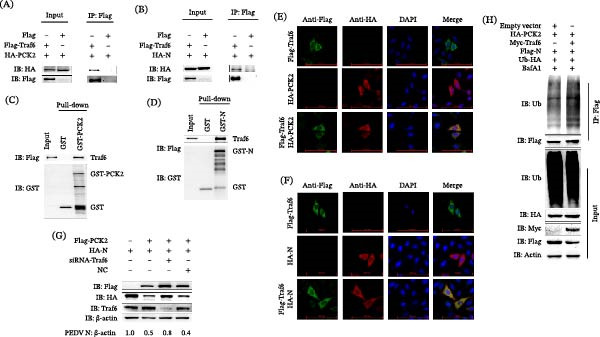
PCK2 recruits Traf6 to degrade PEDV N protein. (A, B) HEK 293T cells were transfected with plasmids encoding Flag‐Traf6, HA‐PCK2, or HA‐N. Co‐IP assay was performed using anti‐Flag conjugated beads, and cell lysates were analyzed by western blotting. (C, D) GST affinity purification assays were used to analyze the interaction between Traf6 and PCK2 or N protein. (E, F) HeLa cells were transfected with plasmids encoding Flag‐Traf6 and HA‐PCK2 or HA‐N, followed by labeling with indicated primary and secondary antibodies. The fluorescence signals were visualized using confocal immunofluorescence microscopy. Scale bars represent 100 μm. (G) HEK 293T cells were cotransfected with plasmids encoding Flag‐PCK2, HA‐N, and Traf6 siRNA, followed by western blotting analysis using an anti‐HA antibody. (H) After cotransfection with HA‐PCK2, HA‐Ub, HA‐Traf6, and Flag‐N, HEK 293T cells were treated with BafA1 and collected. The ubiquitinated N protein was subjected to western blotting after immunoprecipitation with an anti‐Flag antibody.

Proteins destined for proteasomal degradation are typically ubiquitin‐tagged. This process is initiated by the E3 ubiquitin ligase, which facilitates the assembly of polyubiquitin chains on the substrate. These chains function as a degradation tag, enabling recognition by specific shuttle proteins that direct the target to the proteasome [32]. To elucidate how PCK2 promotes autophagic degradation of the PEDV N protein, we employed a screening approach in HEK 293T cells by coexpressing PCK2 with various E3 ubiquitin ligases (Traf6, MARCH8, PARKIN, STUB1, and TRIM21). Coimmunoprecipitation identified Traf6 as a specific interactor with PCK2 (Figure 3A and Figure S1D–G). Further analysis revealed reciprocal interactions, as Traf6 also bound the PEDV N protein (Figure 3B). GST pull‐down assays confirmed that Traf6 directly interacts with both PCK2 and the N protein (Figure 3C,D). Immunofluorescence confocal microscopy revealed that Traf6 colocalizes with PCK2 and the PEDV N protein in the cytoplasm (Figure 3E,F). The above results indicated that Traf6 might play an important role in PCK2‐mediated N protein degradation. In order to verify this speculation, we employed siRNA to knockdown the Traf6 expression in HEK 293T cells and found that inhibiting the expression of Traf6 significantly increased the abundance of HA‐N (Figure 3G). To determine the role of Traf6 in PCK2‐induced autophagic degradation of the N protein, we overexpressed Traf6 in HEK 293T cells. We noted that Traf6 overexpression significantly enhanced the ubiquitination of the N protein (Figure 3H). These findings suggest the function of Traf6 in enhancing the ubiquitination of the N protein during PCK2‐mediated autophagic breakdown.

### 3.4. PCK2 Promotes the Degradation of N via the Traf6‐Tollip‐Autophagosome Pathway

**Figure 4:**
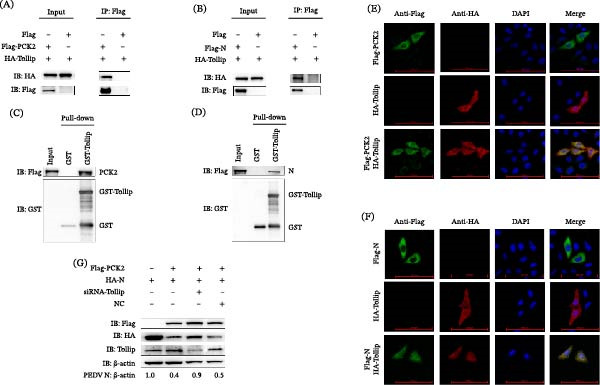
PCK2 facilitates N degradation via the Traf6‐Tollip‐autophagosome pathway. (A, B) HEK 293T cells were transected with plasmids encoding HA‐Tollip and Flag‐PCK2 or Flag‐N. Co‐IP assay was performed using anti‐Flag beads. (C, D) GST pull‐down assay was conducted to investigate the interactions between Tollip and PCK2 or N protein. (E, F) HeLa cells were cotransfected with HA‐Tollip and Flag‐PCK2 or Flag‐N plasmids and incubated with indicated antibodies. The cells were observed by confocal immunofluorescence microscopy. (G) HEK 293T cells were cotransfected with Flag‐PCK2, HA‐N, and Tollip siRNA, followed by western blot to assess N protein levels.

The selectivity of autophagy is fundamentally reliant on diverse cargo receptors, which orchestrate the sequestration of specific cellular components—including damaged organelles and protein aggregates—into autophagosomes for subsequent lysosomal breakdown [33]. To delineate the mechanism of PCK2‐mediated autophagic degradation of the PEDV N protein, we investigated the role of the cargo receptor Tollip. Co‐IP assays confirmed interaction between HA‐Tollip and both Flag‐PCK2 and Flag‐N (Figure 4A,B and Figure S1H, I), which was further validated as direct binding by GST pull‐down assays (Figure 4C,D). Immunofluorescence microscopy demonstrated cytoplasmic colocalization among these proteins (Figure 4E,F). Crucially, siRNA‐mediated knockdown of Tollip substantially impaired PCK2‐induced degradation of the N protein (Figure 4G). These findings collectively indicate that PCK2 suppresses PEDV replication via a PCK2‐Traf6‐Tollip‐autophagosome pathway.

## 4. Discussion

Continuous genetic evolution has driven the emergence of novel, highly virulent PEDV strains, posing severe threats to the global swine industry. Existing vaccines, including inactivated and attenuated versions, provide only suboptimal efficacy against these variants [31, 34, 35]. A deeper understanding of virus‐host interactions is therefore essential to facilitate the development of effective countermeasures against PEDV.

PCK2, also known as PEPCK2 or PEPCK‐M, is encoded by a gene on chromosome 14q11.2‐q12 and gives rise to a 640‐amino acid protein. First isolated from chicken liver, PCK2 is widely expressed across tissues and resides in the mitochondria. Its well‐established functions center on gluconeogenesis, but emerging evidence has linked it to metabolic reprogramming, cancer cell plasticity, and tumor progression [28, 36]. In contrast, whether PCK2 participates in viral infection has remained largely unexplored.

**Figure 5:**
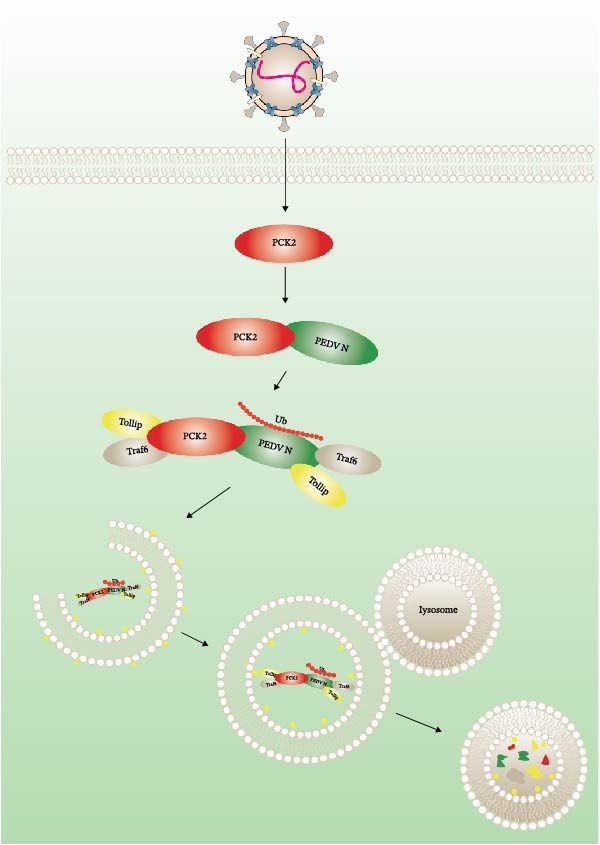
PCK2 inhibits porcine epidemic diarrhea virus replication by promoting viral N protein degradation via autophagy.

As a vital host defense mechanism, autophagy maintains cellular homeostasis through a multistep process involving initiation, M closure, maturation/fusion, and eventual degradation [30, 37, 38]. During PEDV infection, this pathway is activated, as evidenced by LC3‐I to LC3‐II conversion. In selective autophagy, cargo receptors bridge ubiquitinated substrates to LC3‐labeled autophagosomes for degradation [39, 40]. Prior research established that host proteins like BST2 and PABPC4 can exploit this system, utilizing E3 ligase MARCH8 and receptor NDP52 to degrade the PEDV N protein and suppress replication [23, 41]. Based on these findings, our study further demonstrates that PCK2 also recruits the E3 ligase Traf6. Through the interaction between PCK2 and Traf6, the N protein is ubiquitinated, which promotes the binding of Tollip to autophagy‐related proteins (Figure 5). This process facilitates autophagosome maturation and trafficking to achieve protein degradation. This mechanism significantly reduces the intracellular accumulation of the PEDV N protein, thereby inhibiting viral replication. Knockdown of Traf6 or Tollip markedly suppresses autophagosome formation, leading to impaired degradation of the PEDV N protein and enhanced viral replication. Therefore, PCK2 plays a critical role in the degradation of the PEDV N protein and the suppression of viral replication through the PCK2‐Traf6‐Tollip‐autophagosome pathway.

In this study, we uncovered an unexpected antiviral role for PCK2. Overexpression of PCK2 markedly inhibited PEDV replication, and subsequent analysis of its interaction with viral proteins revealed specific binding to the viral N protein. These observations expand the functional landscape of PCK2 beyond metabolism and place it within the realm of antiviral innate immunity. Mechanistically, we found that PCK2 facilitates the selective autophagic degradation of the PEDV N protein by engaging the E3 ubiquitin ligase Traf6 and the cargo receptor Tollip. These components collectively form a functional axis in which PCK2, Traf6, and Tollip cooperate to direct the viral N protein toward autophagic clearance. This represents a previously uncharacterized host strategy that repurposes a metabolic enzyme for antiviral defense.

Precisely due to the limited number of reports on the antiviral role of PCK2, several questions remain to be addressed: The regulatory mechanisms controlling PCK2 expression and activation during PEDV infection are still unclear. In addition, in vivo evidence will be essential to determine whether activating this pathway confers protection against PEDV infection in animals [27]. Addressing these issues will be a critical step toward translating these findings into clinical or practical applications.

## Author Contributions

Ning Kong, Shuang Xue, and Guangzhi Tong conceived, designed, and supervised this study. Ao Gao, He Sun, and Xinyu Yang performed sample collection and experimental studies. Ao Gao, Xinyu Yang, Wenzhen Qin, Yuchang Liu, Zongyan Chen, and Dongfang Zheng participated in data analyses. Ning Kong and Lanlan Zheng contributed reagents/materials/analysis. Ning Kong and Lanlan Zheng provided funding for this study. Ao Gao wrote the first version of the manuscript. The manuscript was revised and edited with contributions from all authors.

## Funding

This work was supported by the Shanghai Agricultural Science and Technology Innovation Program (Grant K2024001), the National Natural Science Foundation of China (Grant 32302891), the Science and Technology Innovation Leading Talent Support Program of Henan Province (Grant 264200510045), the Outstanding Young Innovation Research Group Project of Natural Science Foundation of Henan Province (Grant 262300421002), and the Young Top‐Notch Talents Foundation of Henan Agricultural University.

## Disclosure

All authors proofread and approved the final version of the manuscript.

## Ethics Statement

No animal or human subjects were involved in this study. All cell lines and viral manipulations were performed in compliance with standard biosafety regulations.

## Conflicts of Interest

The authors declare no conflicts of interest.

## Supporting Information

Additional supporting information can be found online in the Supporting Information section.

## Supporting information


**Supporting Information** Figure S1: PCK2 shows no detectable interaction with the PEDV structural protein S1, nor with a subset of the tested E3 ubiquitin ligases and cargo receptors. (A) Flag‐PCK2 does not interact with PEDV S1 protein. Coimmunoprecipitation assays were performed in cells coexpressing Flag‐PCK2 and HA‐tagged PEDV S1. Immunoblotting (IB) with an anti‐HA antibody showed no detectable interaction between Flag‐PCK2 and HA‐S1, while input controls confirmed the expression of both proteins. (B, C) Overexpression of PCK2 did not reduce the protein levels of S2 or E. (D–I) Among the E3 ubiquitin ligases and cargo receptors examined, only Traf6 and Tollip were found to interact with PCK2 in coimmunoprecipitation assays. Coimmunoprecipitation assays were performed in HEK 293T cells cotransfected with Flag‐PCK2 and either MYC or HA‐tagged E3 ubiquitin ligases (MARCH8, PARKIN, STUB1, and TRIM21) (related to Figure 3) or cargo receptors (NDP52 and P62) (related to Figure 4). Cell lysates were immunoprecipitated using anti‐Flag magnetic beads and analyzed by Western blotting.

## Data Availability

The data that support the findings of this study are available from the corresponding author upon reasonable request.
